# Prediction of biological age using machine learning

**DOI:** 10.1371/journal.pone.0330184

**Published:** 2025-09-24

**Authors:** Kai Zhang, Po-Chung Chen, YiYang Huang, Shiow-Jyu Tzou, Sheng-Tang Wu, Ta-Wei Chu, Chung-Che Wang, Jyh-Shing Roger Jang

**Affiliations:** 1 Yiwu Industrial and Commercial College, Yiwu, Zhejiang, China; 2 Graduate Institute of Network and Multimedia, National Taiwan University, Taipei, Taiwan; 3 Division of Family Medicine, Taoyuan Armed Forces General Hospital, Taoyuan, Taiwan; 4 Department of Computer Science and Information Engineering, National Taiwan University, Taipei, Taiwan; 5 Teaching and Researching Center, Kaohsiung Armed Forces General Hospital, Kaohsiung, Taiwan; 6 Institute of Medical Science and Technology, National Sun Yat-sen University, Kaohsiung, Taiwan; 7 Division of Urology, Department of Surgery, Tri-Service General Hospital, National Defense Medical Center, Taipei, Taiwan; 8 Division of Urology, Department of Surgery, Kaohsiung Armed Forces General Hospital, Kaohsiung, Taiwan; 9 Department of Obstetrics and Gynecology, Tri-Service General Hospital, National Defense Medical Center, Taipei, Taiwan; 10 MJ Health Screening Center, Taipei, Taiwan; Covenant University, NIGERIA

## Abstract

In response to Taiwan’s rapidly aging population and the rising demand for personalized health care, accurately assessing individual physiological aging has become an essential area of study. This research utilizes health examination data to propose a machine learning-based biological age prediction model that quantifies physiological age through residual life estimation. The model leverages LightGBM, which shows an 11.40% improvement in predictive performance (R-squared) compared to the XGBoost model. In the experiments, the use of MICE imputation for missing data significantly enhanced prediction accuracy, resulting in a 23.35% improvement in predictive performance. Kaplan-Meier (K-M) estimator survival analysis revealed that the model effectively differentiates between groups with varying health levels, underscoring the validity of biological age as a health status indicator. Additionally, the model identified the top ten biomarkers most influential in aging for both men and women, with a 69.23% overlap with Taiwan’s leading causes of death and previously identified top health-impact factors, further validating its practical relevance. Through multidimensional health recommendations based on SHAP and PCC interpretations, if the health recommendations provided by the model are implemented, 64.58% of individuals could potentially extend their life expectancy. This study provides new methodological support and data backing for precision health interventions and life extension.

## Introduction

### Research background

**Fig 1:**
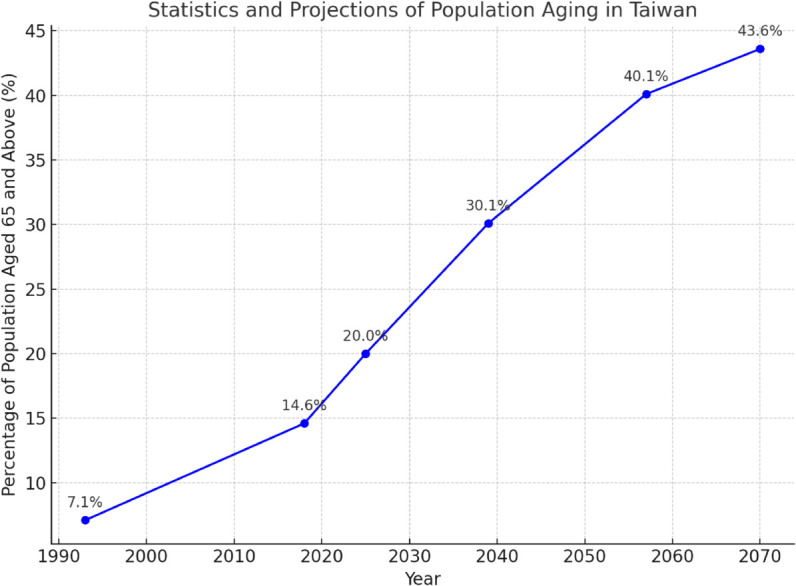
Statistics and projections of population aging in Taiwan.

According to the content published by the National Development Council regarding the issue of population aging in Taiwan, the proportion of elderly individuals reached 14% of the total population in 2018, marking the transition into an aging society. It is projected that Taiwan will enter a super-aged society by 2025 [1]. The detailed data is shown in Fig 1.

At the same time, Taiwan has made significant advancements in personalized medicine, with the government recently providing momentum for commercial investment and research efforts in precision medicine [2]. Advanced medical research institutions in Taiwan, such as Taipei Veterans General Hospital, have also established precision medicine teams, aiming to create a genomic database for Taiwan and identify common disease risk factors among the Taiwanese population [3].

In recent years, with advancements in precision medicine technology and an overall increase in public health awareness, alongside challenges such as population aging, research on biological age has become increasingly relevant. Unlike the concept of chronological age, which is solely related to the time elapsed since birth, biological age assesses an individual’s current physiological state, thereby providing a more accurate description of their physical aging status.

Accurate assessment of biological age offers several benefits in addressing the issue of population aging. It allows for a more precise evaluation of the aging process; in fact, aging should more accurately be viewed as physical degeneration. Thus, using biological age to classify older individuals is more precise than relying on chronological age. Additionally, research on biological age enhances our understanding of the biological mechanisms underlying aging, providing guidance for the public in mitigating aging effects and helping to alleviate the intensifying challenges of population aging.

The development of precision medicine lays a solid foundation for constructing more accurate biological age models. Aging is a multifactorial process, and precision medicine offers a wealth of information on genomics, metabolites, and other aspects for individuals, aiding in the construction of biological age models related to aging. Moreover, precision medicine emphasizes personalization, which aligns with the objective of constructing biological age models tailored to individual aging characteristics, thereby facilitating the concept of personalized healthcare.

### Research contribution

The contributions of this study are as follows:

- **Proposed a machine learning-based biological age prediction model**: Using health examination data and the LightGBM model combined with residual life estimation, the study achieved a quantitative analysis of individual physiological aging, with significant improvements in prediction accuracy compared to traditional methods.
- **Optimized data imputation methods to enhance prediction accuracy**: By employing the MICE imputation method for handling missing data, the model’s prediction accuracy was significantly improved, with a 23.35% increase in performance compared to the data filtering method.
- **Validated biological age as an effective metric for quantifying health status**: Through Kaplan-Meier survival analysis, the model demonstrated a strong capability to distinguish between different health levels, affirming the feasibility and accuracy of biological age as an indicator for assessing individual health conditions.
- **Provided personalized health management recommendations**: Using SHAP and PCC interpretation tools, the model identified critical aging biomarkers and generated health recommendations for slowing aging. The experimental results indicate that applying these recommendations could potentially extend the expected lifespan of 64.58% of individuals, offering data support and practical value for future precise health interventions.

### Section overview

This paper is divided into six sections:

1. The first section is the introduction, which presents the research background, motivation, and outline of the paper.
2. The second section is the literature review, which discusses existing studies on biological age prediction, covering both traditional biological methods and machine learning-based approaches.
3. The third section introduces the dataset, detailing the dataset from MJ Health Screening Center used in this study.
4. The fourth section describes the research methodology, outlining how to construct a regression model for residual life prediction and how biological age is subsequently estimated. It also includes the validation process for the prediction model and the provision of interpretability.
5. The fifth section focuses on experimental design and results discussion, explaining the design of the experiments and the corresponding results, along with an analysis of the findings.
6. The sixth section presents the conclusion and future work, summarizing the research and outlining potential future research directions.

## Related work

This section will focus on reviewing relevant literature related to this research. It will first introduce the literature concerning human aging and then discuss the literature on biological age prediction. The biological age prediction will be categorized into two approaches: predictions from a traditional biological perspective and predictions utilizing machine learning methods.

### Human aging

Aging is a naturally occurring biological process that results from the interplay of multiple factors, and biological age is used to measure the level of aging in the human body. The aging process is associated with changes in various aspects of the body, including:

- Aging can lead to brain-related pathologies, including protein-related diseases such as Alzheimer’s disease and tauopathies. These aging-related conditions often result in cognitive impairment issues [4].
- Aging also causes problems in the neuromuscular system; as aging progresses, the functionality of the neuromuscular system declines, leading to increased fascial stiffness, reduced fascial elasticity, decreased strength, and diminished coordination [5].
- The aging process also induces changes in the structural functions of the skin, including thinning of the epidermis, atrophy of the dermis, and a weakened immune inflammatory response [6].
- The effects of aging also extend to the internal organs. For instance, the kidneys experience a reduction in volume and an increase in roughness, leading to increased levels of kidney sclerosis and a decrease in the number of glomeruli [7].

### Biological age prediction from a traditional biological perspective

Predicting biological age from a biological perspective has traditionally been one of the most direct approaches in this field of research. Traditional biological methods for predicting biological age often rely on various biomarkers, such as [8]:

- Telomere Length: Telomeres shorten with each cell division, making telomere length a potential indicator for predicting biological age.
- Transcriptome: Biological age can be predicted through gene expression patterns.
- Proteome: Biological age prediction is achieved by studying the protein patterns in the body.
- Metabolomics: Biological age is estimated based on the metabolic profile of the body.
- Epigenetic Clocks: Biological age prediction is made by measuring DNA methylation patterns.
- Composite Biomarkers: This approach combines multiple biomarkers to predict biological age.

Among these methods, predicting biological age based on epigenetic clocks shows considerable promise for success.

### Biological age prediction based on machine learning

In recent years, with the extensive and in-depth application of machine learning methods across various fields, from respiratory diagnostics [24] to finance and autonomous driving, research on biological age prediction has also emerged based on machine learning algorithms. Unlike biological approaches that often rely on specific biomarkers, machine learning can utilize multiple biomarkers to jointly assess biological age. It is noteworthy that most current literature on machine learning-based biological age prediction uses chronological age as the target for training and prediction.

**Fig 2:**
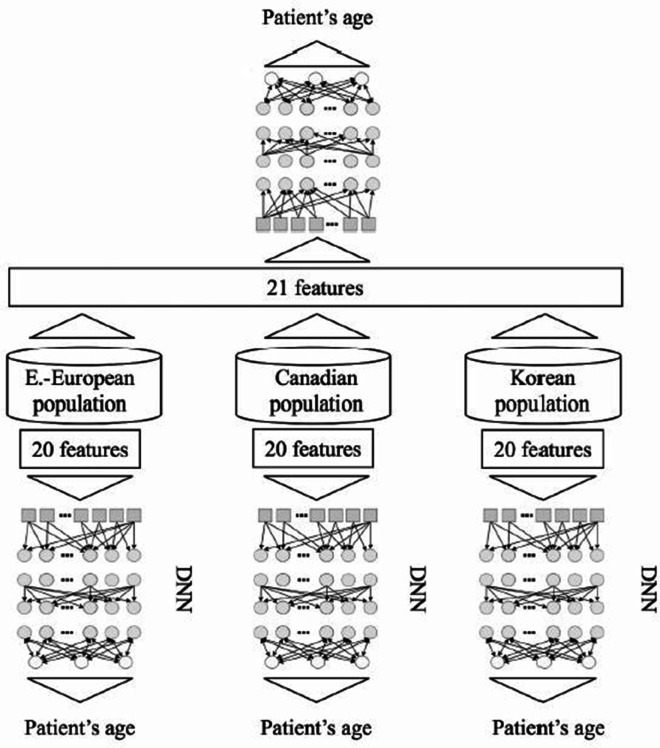
DNN-based biological age prediction architecture for different populations [9].

In [9], the authors developed a biological age prediction model based on deep learning. For different populations, including South Korean, Canadian, and Eastern European groups, biochemical markers were used to train a deep neural network to predict biological age. The classification of populations was included as a feature in the training, which expanded the training data and constructed a model capable of predicting biological age across various populations, as shown in Fig 2.

**Fig 3:**
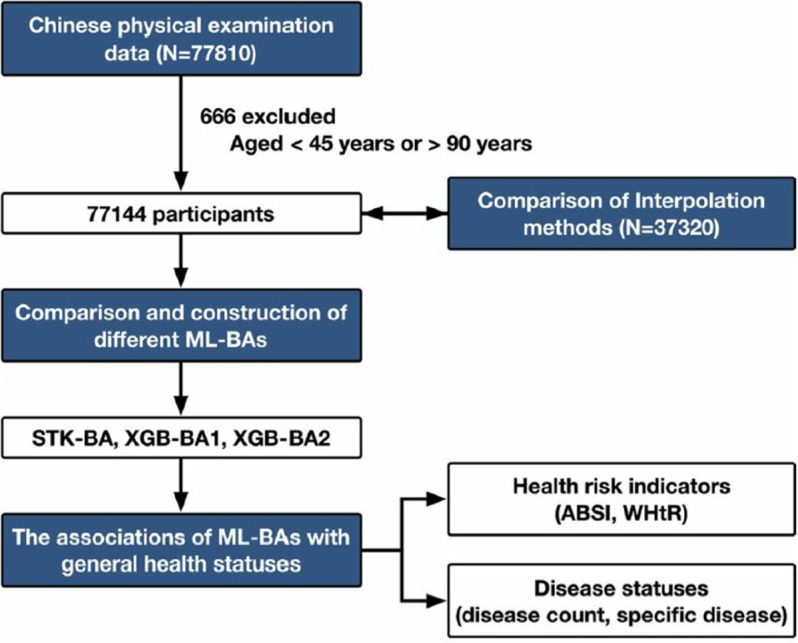
Biological age prediction architecture based on traditional machine learning methods [9].

In [10], the authors constructed a biological age prediction model based on traditional machine learning. The dataset used in this study was derived from health examination data of the population in Zhejiang Province, China, where individuals aged between 45 and 90 were selected for analysis. Using chronological age as the target for prediction, machine learning algorithms such as XGBoost (eXtreme Gradient Boosting) were employed to build the biological age prediction model. Finally, the predicted biological age was validated against various health risk indicators, such as WHtR (Waist-to-Height Ratio), as illustrated in Fig 3.

### Summary

To systematically compare existing studies on machine learning-based biological age prediction, we have summarized key literature in Table 1. This table outlines the methodology, dataset, as well as the primary strengths and weaknesses of each approach, providing a clear overview of the current research landscape.

**Table 1:** Comparison of related studies and this work.

Study	Methodology	Dataset	Strength	Weakness
Mamoshina et al. [9]	Deep Neural Network (DNN)	Biochemical indicators from populations in Korea, Canada, Eastern Europe, etc.	- Integrated data from multiple populations to build a cross-population prediction model.	- Chronological age was still used as the prediction target.
Yang et al. [10]	Traditional machine learning (e.g., XGBoost)	Health checkup data of people aged 45–90 in Zhejiang, China	- Validated predicted biological age against health risk indicators (e.g., WHER).	- Chronological age used as prediction target. - Age range of participants was limited (45–90 years).
This Study	LightGBM with residual life prediction	MJ Health dataset (Taiwan), including death follow-up data	- Directly predicts residual life, more reflective of physiological aging. - MICE imputation improved model performance. - Biological age validated through K-M survival analysis.	- Small sample size in “Young” female group affected K-M analysis. - PCC method used for interpretation cannot handle categorical variables.

## Dataset

This section will introduce the dataset utilized in this research. The dataset is licensed from the MJ Health Screening Center and primarily consists of two parts: one part records the data obtained during health examinations of individuals, while the other part contains follow-up data on the mortality status of these individuals.

### Basic information

The content of this dataset is derived from the standard medical health examination items of the MJ Health Management Agency, recorded from 1998 to 2017. Participants in the health examination are required to complete three aspects:

1. Self-administered questionnaires, which include various surveys regarding lifestyle habits, such as alcohol consumption, surgical history, and past experiences.
2. Measurement of basic physical information, including height, weight, and BMI.
3. Laboratory tests, including blood and urine analyses.

After collection, each health examination record contains information from the aforementioned three aspects, totaling 456 fields. The total number of health examination records amounts to 1,302,799, with 647,546 records for males and 655,253 records for females.

#### Distribution of health examination years.

**Fig 4:**
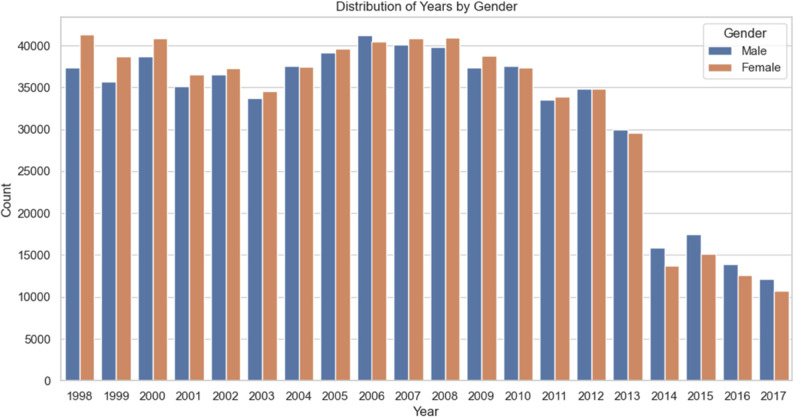
Distribution of health examination years.

According to Fig 4, there was not much change in the number of health examinations from 1998 to 2013, and the gender ratio remained approximately the same. However, the decline in the total number of health examinations starting in 2013 is attributed to the Taiwanese government’s tightening of personal data collection regulations, which restricted the gathering of such information.

#### Age distribution of health examination participants.

**Fig 5:**
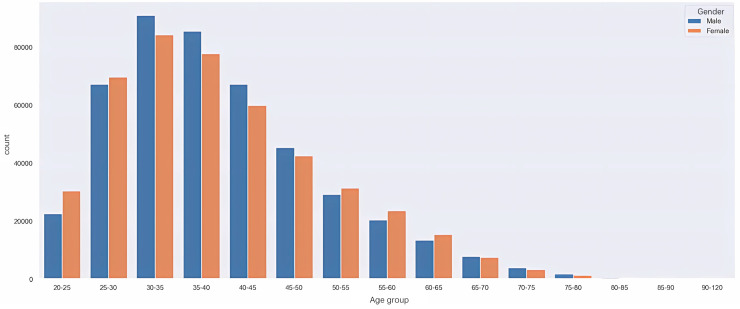
Age distribution of health examination participants.

According to Fig 5, the proportions of males and females in different age groups within the health examination records are approximately the same. Among these age groups, the majority consists of young and middle-aged individuals aged 25 to 45.

### MJ health examination participant mortality follow-up dataset

This dataset tracks the mortality status of individuals who participated in the MJ health examination program up to November 30, 2018. The mortality information is obtained from the MJ Health Screening Center in collaboration with government departments in Taiwan.

The dataset includes the de-identified ID of health examination participants, the number of times they attended health check-ups, the date of death (left blank if not deceased), and the cause of death (also left blank if not deceased). It encompasses mortality information for all participants in health examinations from the start of data collection in 1998 until 2018, totaling 1,302,799 records (corresponding to the number of health examination visits).

### Example and significance of the merged dataset

We merged the two datasets, aligning the mortality information with the health examination data of the individuals while they were alive. The resulting data is presented in Table 2.

**Table 2:** Sample of a single record from the merged dataset.

Pid	Physical Examination Date	Physical Examination and Questionnaire Content	Date of Death	Cause of Death
c19b000001	2010/12/01	(456 values)	2018/05/01	malignant tumor

In subsequent research, this study will consider using health examination data and questionnaire data of examinees as feature values, and by calculating their time of death, obtain the actual remaining lifespan at the time of the examination. The work in later sections will focus on selecting methods or building a model to predict the relationship between health examination and questionnaire data with remaining lifespan, aiming to identify an effective prediction model and further analyze the influence of each feature on remaining lifespan.

## Research methods

This study was conducted under the supervision of the “Institutional Review Board of Kaohsiung Armed Forces General Hospital” (Approval No.: KAFGHIRB 111-015) to ensure complete anonymity of all data. The original health examination data were maintained by the MJ Health Research Foundation. This study utilized secondary data collected between January 2010 and December 2017 and did not involve participant recruitment. All participants provided informed written consent, and for minors, consent was obtained from their parents or guardians. The data used in this study were accessed on December 19, 2022, and all were anonymized. Throughout the study, participant confidentiality was strictly protected in compliance with all ethical guidelines and regulations governing human research.

This section will introduce the methods used throughout the research process, including the basic data processing steps and the approach to obtaining a complete dataset. It will also cover the machine learning algorithms employed for model construction, as well as the methods for model evaluation and interpretation.

### Dataset record and feature selection

Since this study is a retrospective analysis using an existing database from the MJ Health Research Foundation, the sample size was not determined through a priori power analysis. Instead, it was derived by applying systematic inclusion and exclusion criteria to the initial 1,302,799 health screening records. The primary purpose of this filtering process was to construct a suitable and unbiased dataset for training a model aimed at predicting remaining lifespan based on the natural aging process. It is important to note that the initial cohort was based on a non-probability, convenience sampling method, as it comprised individuals who voluntarily participated in health screenings at a specific institution, rather than being randomly selected from the general population. The specific screening criteria are detailed as follows.

In the first step, we filter out biased data from the merged dataset, which includes removing three types of biased records:

- Records of individuals with accidental deaths: Non-natural causes of death may result in an earlier death than natural causes would, introducing bias if used as a target for the model’s lifespan prediction. This step removed 4,494 records, accounting for 0.38%.
- Records of individuals under 20 years of age: This demographic’s biochemical data may deviate significantly, making it unsuitable for inclusion in the dataset used to build the model. This step removed 30,764 records, or 2.36%.
- Records of long-term medication users: Regular medication use can artificially impact biochemical indicators, so these records were also excluded. This step removed 356,845 records, or 27.4%.

**Fig 6:**
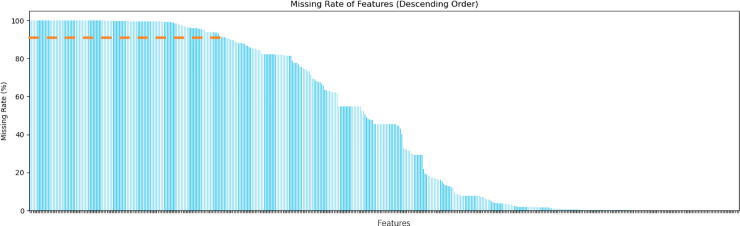
Features with high missing rates filtered out.

In the second step, we remove features with a high rate of missing data. Since the dataset includes questionnaire responses from health examination participants, certain features have high missing rates, making them unsuitable for subsequent data imputation. As shown in Fig 6, we chose to exclude features with a missing rate over 90% (above the orange dashed line in the figure), which led to the removal of 135 features, or 29.3% of the total.

Since our method for constructing biological age relies on predicting the remaining lifespan of health check participants, which can only be obtained from records with a death date, we need to retain only those records that include this information. At this step, 889,774 records are filtered out, accounting for 68.3% of the data.

In the final step, the predicted biological age will be derived from first predicting the remaining lifespan of the health check participants. This remaining lifespan will be obtained by calculating the difference between the health check participants’ examination dates and their recorded death dates. By subtracting the examination date from the death date, we can obtain their remaining lifespan at the time of the health check (measured in years).

In the end, we obtained an effective sample size of 12,460 males and 8,013 females. The dataset contains 325 features, which encompass three types: self-reported questionnaire responses, basic physical information measurements, and laboratory test results. Our prediction target is the remaining lifespan of each health check participant, which is calculated based on the methods described earlier.

However, it is important to clarify that a single individual may have multiple health examination records during the period from participation to death. Each record is treated as a separate instance for calculation. In the subsequent sections, we consider each health examination record as an independent instance for prediction and evaluation. In the article, “male” and “female” are used for distinction, but here, a “male” or “female” refers specifically to a single health examination record of a male or female individual.

### Handling missing values

#### MICE.

**Fig 7:**
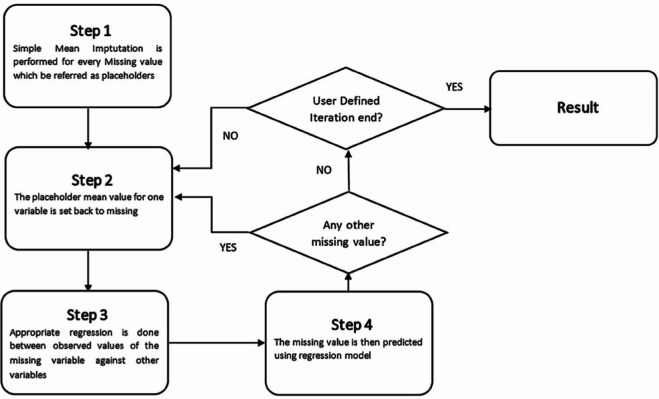
MICE flow chart [13].

In the data preprocessing for this study, one method for obtaining a complete dataset is through imputation algorithms. The chosen imputation algorithm is MICE (Multivariate Imputation by Chained Equations) [11]. MICE is an iterative imputation method that generates a complete dataset by treating missing data as unknown values and refining these estimates through multiple iterations. As shown in Fig 7, the main steps are as follows [12]:

1. Start with a simple imputation, such as mean imputation, to fill in the missing values in the dataset as a preliminary step.
2. Set one of the imputed values back to its missing state, which will become the target for imputation prediction.
3. Use the other records to construct a prediction model specifically for that missing target value.
4. Predict the missing value using the model just built.
5. Repeat steps 2-4 for all features with missing values, so that every missing value is estimated iteratively, completing one cycle.
6. Define the number of cycles, during which missing values are updated with each prediction cycle.

**Fig 8:**
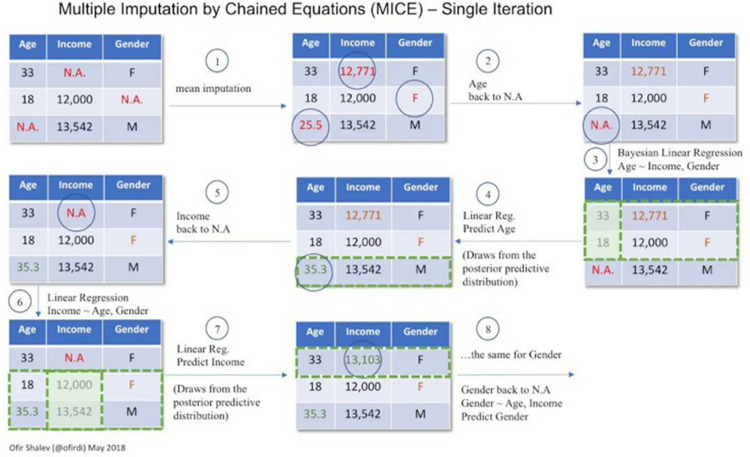
MICE example [11].

Let’s look at how MICE works through a practical example, as illustrated in Fig 8:

1. We start with a dataset containing missing values, as shown in the top left section of the figure, with missing values across three features.
2. We perform mean imputation on each of the three features. For numerical features like Age and Income, the mean value is used. For the categorical feature Gender, we use “F” since M and F appear with equal frequency.
3. We set the missing value in Age, the feature we want to impute more accurately, back to its missing state.
4. Using the complete records (e.g., rows 1 and 2), we build a linear regression model to predict the missing value in Age.
5. Steps 2-4 are repeated for each missing value to predict until all missing values have been imputed.
6. The above constitutes one iteration. The process is repeated for a set number of iterations until stopping.

**Fig 9:**
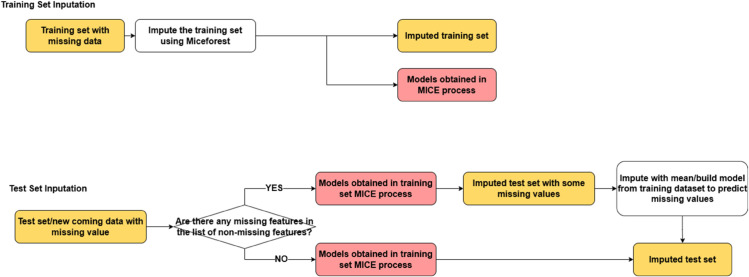
Imputation process.

In this study, we apply imputation to each of the three datasets partitioned in the previous section, as illustrated in the workflow diagram (Fig 9).

- Training Set Imputation Phase: We apply MICE directly to the training set, which completes the imputation and generates a series of models from this process. These models serve as potential tools for imputing the validation and test sets.
- Validation and Test Set Imputation Phase: It’s important to note that there may be features with missing values in the validation or test sets that do not have corresponding missing values in the training set. In such cases, no model exists to impute those features. In MICE’s implementation in the Miceforest package, these features are directly skipped when this issue occurs. When there are no such instances, we simply use the models generated from the training set to complete imputation. When such instances do occur, we perform an initial imputation using the existing models, obtaining partially imputed data. We then train additional models on the training set to fully complete the imputation.

#### Filtering records and features using rules.

The second method used in this study to obtain a complete dataset involves filtering features and records in the dataset to produce a complete dataset. A complete dataset is defined as one without missing values and suitable for model training.

We followed the two filtering steps below to obtain the complete dataset:

1. Remove features with a missing rate greater than 2%. Here, we exclude 181 features, accounting for 55.7% of the total.
2. After filtering in the previous step, we retain only features with relatively low missing rates. This allows us to further remove all records containing any missing values. At this step, we delete 1,442 records, which constitutes 7.05% of the total.

The summary of the complete dataset information after processing is shown in Table 3.

**Table 3:** Table of information for the filtered complete dataset.

Dataset	Feature Number	Record Number (male)	Record Number (female)
Filtered	144	11,714	7312

### Building a machine learning model using LightGBM to predict remaining lifetime

**Fig 10:**
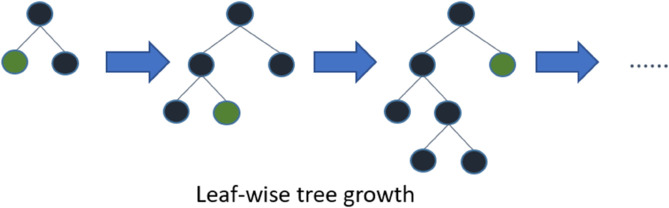
The tree growth diagram of leaf-wise method.

In previous studies, XGBoost was used to predict biological age. However, we found that using the LightGBM model [14] is more suitable for predicting biological age. Compared to XGBoost, LightGBM has several improvements:

- It uses a histogram-based algorithm, which reduces memory usage and decreases the cost of calculating gain during each split.
- It adopts a leaf-wise tree generation method, as shown in Fig 10. Compared to the traditional depth-wise tree generation method, this approach reduces unnecessary node splits and lowers loss. It only splits the leaf node with the highest gain until the stopping condition is met.
- It supports categorical features, eliminating the need for encoding categorical features before training.

### Conversion from remaining life expectancy to biological age

Since our machine learning model is designed to directly predict the residual life span, while our ultimate objective is to estimate biological age, we propose a conversion method from residual life span to biological age as follows:

Bio.age=Avg.Age−Residual Life, (1)

where Bio.age denotes the biological age, Avg.Age represents the average life expectancy of the Taiwanese population, and Residual Life stands for the residual life span predicted for health examination participants.

Eq 1 is based on a fundamental concept: Avg.Age represents the average life expectancy of the Taiwanese population as published by government sources. Subtracting the remaining years until a person’s predicted end of life from this average yields an estimated biological age, which, unlike chronological age, reflects the true state of bodily aging.

**Fig 11:**
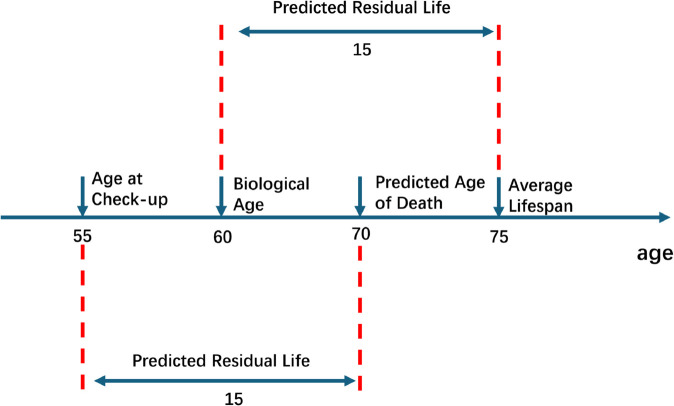
Example of converting residual life to biological age.

For example, as illustrated in Fig 11, consider an individual participating in a health examination with a chronological age of 55. If the model estimates a residual life span of 15 years, this individual is expected to live to 70. If the latest government statistics indicate an average life expectancy of 75 years in this individual’s region, the difference of 5 years between the predicted lifespan of 70 years and the average expectancy of 75 years reflects a relative aging of 5 years beyond the norm. Thus, the biological age would be the chronological age of 55 plus 5, resulting in a biological age of 60. Alternatively, one could directly subtract the estimated residual life of 15 years from the average life expectancy of 75, also arriving at a biological age of 60. Our Avg.Age is derived from government-published data [15,16], with examples of this data shown in Table 4.

**Table 4:** Average life expectancy by city.

City	Year 111		
	Total	Male	Female
**(Unit: Year)**	(1)	(2)	(3)
**All**	79.84	76.63	83.28
**New Taipei**	81.32	78.26	84.39
**Taipei**	83.75	80.55	86.94
**Taoyuan**	81.04	77.93	84.14
**Taichung**	80.37	77.34	83.35
**Tainan**	79.67	76.72	82.57
**Kaohsiung**	79.47	76.56	82.43
**Keelung**	78.40	74.83	81.96
**Hsinchu City**	81.96	78.83	85.08
**Hsinchu County**	80.50	77.65	83.36
**Miaoli**	78.89	75.66	82.12
**Changhua**	78.70	75.48	81.89
**Nantou**	78.57	75.50	81.61
**Yunlin**	77.66	74.39	80.93
**Chiayi City**	78.56	75.59	81.56
**Chiayi County**	77.53	74.02	80.95
**Pingtung**	78.50	75.43	81.60
**Yilan**	78.07	74.65	81.43
**Hualien**	75.45	71.84	79.07
**Taitung**	74.08	70.04	78.17
**Penghu**	78.70	75.27	82.23
**Kinmen**	81.72	78.36	85.30
**Lienchiang**	80.99	77.47	84.10

It is worth noting that the published government data only covers years from 2010 (ROC year 99) to 2022 (ROC year 111), and does not include records for Matsu. In our mortality tracking data, the distribution of death years ranges from ROC year 87 to 107. Therefore, for subsequent model validation, if we cannot locate an average life expectancy corresponding to the specific year and region of a health examination record, we will temporarily use the latest available national average life expectancy for the respective gender (currently the year 2022) as the value for Avg.Age.

### Evaluation methods

After predicting the remaining life using the machine learning model and converting the remaining life to biological age, we need to evaluate the biological age proposed above. Due to the nature of biological age, there is no direct objective true value for evaluation. Therefore, survival analysis tools are typically used for indirect assessment. In this study, the survival analysis method employed to evaluate the accuracy of the proposed biological age is the Kaplan-Meier estimator (K-M estimator) [17].

The K-M estimator is an estimate of the survival function, which describes the probability of a system remaining alive after a certain period of time. In this context, it represents the probability of a health check participant surviving for a given time after the checkup. The formula for the K-M estimator is defined as follows:

$$\hat{S}(t)=\prod_{i:t_{i}<t}\left(1-\frac{d_{i}}{n_{i}}\right),$$ (2)

Where *t*_*i*_ is the time at which at least one event (in this study, death) occurs, *d*_*i*_ is the number of deaths at time *t*_*i*_, and *n*_*i*_ is the number of people still alive at time *t*_*i*_.

**Fig 12:**
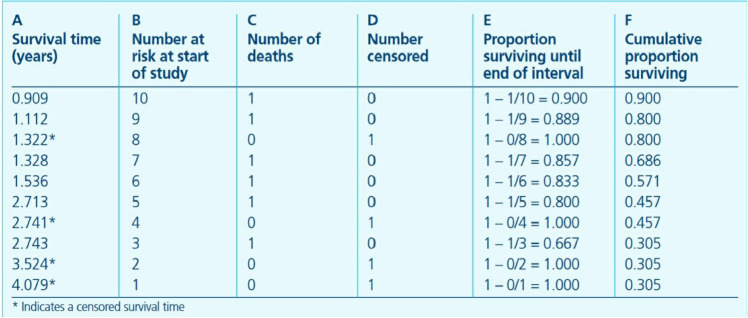
Survival analysis data example [18].

For a dataset as shown in Fig 12, which records the number of deaths at each time point, the K-M estimator for the survival function can be constructed based on the formula in 2.

We will perform biological age prediction on the test set. After obtaining the biological age, we will compare it with the chronological age and categorize the population into three corresponding groups: the aging group, representing individuals with a more aged body; the stable group, representing those with normal aging; and the young group, representing individuals with a relatively younger body. Additionally, we set a tolerance range of 2.5 for the stable group, as shown in Formula 3.

Aginggroup:BioAge>Chronological Age+2.5 (3)

Stablegroup:|BioAge−Chronological Age|≤2.5 (4)

Younggroup:BioAge<Chronological Age−2.5 (5)

Next, we will construct the K-M Curve for each of the three groups. According to our expectations, the biological age we propose will correctly classify the aging status of the population based on Formula 3. Specifically, among the three K-M Curves, the young group should have the highest survival probability, followed by the stable group and then the aging group.

### Statistical analysis

Descriptive statistics were used to summarize the baseline characteristics of the study participants. Continuous variables were expressed as mean ± standard deviation (SD), while categorical variables were presented as frequencies and percentages (%).

All statistical analyses were conducted using Python along with relevant scientific libraries. The performance of the residual life prediction model was evaluated using the coefficient of determination (R^2^), mean absolute error (MAE), mean squared error (MSE), and root mean squared error (RMSE).

To validate the biological age metric, participants in the test set were categorized into three groups—Aging, Stable, and Young—based on the difference between their biological and chronological ages. Kaplan-Meier survival curves were generated for each group, and differences in survival distributions were assessed using the log-rank test. The log-rank test assumes non-informative censoring.

For model interpretability, the Pearson correlation coefficient (PCC) was used to assess linear relationships between continuous biomarker variables. A p-value of less than 0.05 was considered statistically significant for all hypothesis tests.

### Ways to interpret models

This section will introduce methods for interpreting the model, primarily SHAP (SHapley Additive exPlanations) [19] and a combination of SHAP with the Pearson Correlation Coefficient (PCC) [20]. In this study, these model interpretation methods serve to explain to health check-up participants the contributing factors in their biological age prediction and to highlight areas for potential improvement, offering guidance on actions that may help slow the aging process.

$$g(z^{\prime })=\phi_{0}+\sum_{i=1}^{M}\phi_{i}z_{i}^{\prime },$$ (6)

In Eq 6, $z^{\prime }\in \{0,1{\}}^{M}$ represents whether a feature *i* is available (in this study, $z^{\prime }$ is always 1), where *M* denotes the total number of features. The term $\phi_{i}$ represents the contribution of feature *i* to the predicted value, while $\phi_{0}$ represents the model’s base value (which corresponds to the mean prediction value, *E*[*f*(*x*)], across the training data). This formula embodies the additive property of Shapley values, indicating that the final prediction is the sum of contributions from each individual feature.

**Fig 13:**
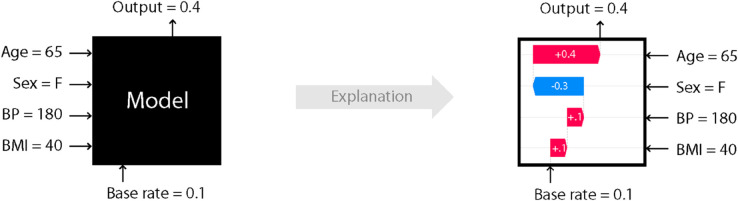
Illustration of SHAP.

As illustrated in Fig 13, we start with an input data instance that includes four features, such as Age, along with their respective values. We have a model to be explained, where we know the model’s average output for the training data is 0.1 and the output value for this specific input data is 0.4. Using SHAP, we can understand why this input yields an output of 0.4. After applying SHAP, we obtain the contribution from each feature. By adding the base rate (average output) to the contributions of the four features, we arrive at the output value of 0.4.

### SHAP combined with PCC

The Pearson Correlation Coefficient [20], often simplified as the correlation coefficient, is used to measure the linear relationship between two continuous variables. The formula is defined as follows in 7:

$$r_{xy}=\frac{\sum_{i=1}^{n}(x_{i}-\bar{x})(y_{i}-\bar{y})}{\sqrt{\sum_{i=1}^{n}(x_{i}-\bar{x}{)}^{2}}\sqrt{\sum_{i=1}^{n}(y_{i}-\bar{y}{)}^{2}}},$$ (7)

In Eq 7, *n* represents the sample size, *x*_*i*_ and *y*_*i*_ are the *i*-th individual samples, $\bar{x}$ is the mean of *x*, and $\bar{y}$ is the mean of *y*.

By combining Pearson Correlation Coefficient (PCC) with SHAP, we aim to offer health check participants more targeted recommendations for anti-aging efforts. The combination process follows these steps:

1. Calculate the PCC among continuous variables in our dataset.
2. Use SHAP analysis to identify the top three aging factors impacting the health check participant.
3. For each of these top three aging factors, extract the three most correlated factors based on PCC.
4. Present the aging factors and their correlated variables as potential modifiable anti-aging directions, enabling medical experts to provide informed recommendations.

**Fig 14:**
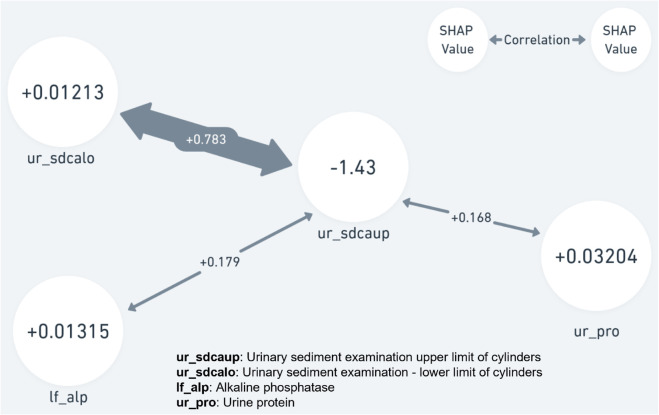
PCC combined with SHAP.

As illustrated in Fig 14, suppose that the central feature, *ur*_*s*_*dcaup* (urinary sediment examination - upper limit of cylinders), is one of the top three aging factors. Based on the pre-calculated PCC values, the three most correlated features are *ur*_*s*_*dcalo* (urinary sediment examination - lower limit of cylinders), *ur*_*p*_*ro* (urine protein), and *lf*_*a*_*lp* (alkaline phosphatase). These four features are then provided to a medical professional, who can offer the health check participant tailored recommendations for improvement.

## Experiment design and results discussion

This section will introduce the context of the experiment design and the corresponding experimental results. The experiment is broadly divided into three parts: the construction of the biological age prediction model, the evaluation of the proposed biological age, and the explainability of the proposed biological age.

### Overall experimental process

In our study, the primary experiment is the biological age prediction experiment. We tested different data imputation methods and machine learning models to identify the most suitable method for biological age prediction, resulting in a well-performing model.

Subsequently, in the biological age assessment experiment, we evaluated the validity of our biological age prediction model using the Kaplan-Meier estimator.

In the exploratory experiment on features influencing biological age, we analyzed the model to identify the factors most influential in predicting biological age. We compared these findings with previous studies and Taiwan’s top ten causes of death, validating the effectiveness of our approach while providing new medical recommendations to focus on.

Finally, we conducted an experiment exploring the impact of healthcare recommendations on individuals. In this experiment, we hypothesized that individuals who followed doctors’ recommendations to improve certain health conditions would experience an increase in their lifespan.

### Experimental setup

#### Experimental environment.

The hardware environment of the experiment is shown in Table 5, and the software versions are listed in Table 6.

**Table 5:** Hardware environment table.

Hardware	Configuration
CPU	AMD Ryzen 9 5900HS
GPU	NVIDIA GEFORCE RTX3060
RAM	32GB

**Table 6:** Software environment table.

Software	Version
Python	3.10.11
Scikit-learn	1.2.2
LightGBM	3.3.5
XGBoost	1.7.3
Miceforest	5.6.2
Hyperopt	0.2.7
Lifelines	0.27.7
Pandas	2.0.1

#### Dataset splitting.

This section will introduce the method for splitting the dataset into training, validation, and test sets.

The split ratio is as follows: 80% of the data is used for the training set, 10% for the validation set, and the remaining 10% for the test set. It is important to note that since our residual life prediction model is highly correlated with age, we aim to maintain consistent age group distributions across all three sets.

To achieve this, we first categorize each record in the dataset into one of three age groups: 20-40, 41-60, and 61-120. Then, using the ‘train_test_split‘ method from scikit-learn [21], we set the parameter ‘stratify‘ to be our age groups. This ensures that the distribution of age groups in the three splits remains consistent.

After splitting the dataset, a summary of the resulting sets is shown in Table 7 and Table 8.The data in Table 7 represent the complete dataset obtained through MICE imputation, while the data in Table 8 represent the complete dataset obtained using the filtering method.

**Table 7:** Complete dataset obtained by imputation.

Set (Proportion)	Number of Features	Male	Female
Training Set (80%)	325	9968	6411
Validation Set (10%)	325	1246	801
Test Set (10%)	325	1246	801

**Table 8:** Complete dataset obtained by filtering.

Set (Proportion)	Number of Features	Male	Female
Training Set (80%)	144	9372	5850
Validation Set (10%)	144	1171	731
Test Set (10%)	144	1171	731

#### Experimental parameter settings.

This section presents the model parameter settings used in the experiment. The model used is LightGBM. Since two different methods were employed for handling missing values in the dataset, different model parameters will be used for each method and presented in two separate tables. In the initial training experiments, we selected the parameters that achieved better training results for each method. The model parameters for the method using MICE imputation are shown in Table 9, and the model parameters for the method using the filtering approach to remove records with missing values are shown in Table 10.

**Table 9:** Model parameters for biological age prediction with MICE imputation.

parameter	male	female
lambda_l1	1	10
lambda_l2	92	90
learning_rate	0.1581	0.1011
max_depth	6	11
min_child_weight	5	1
min_data_in_leaf	50	130
n_estimators	2825	3850
num_leaves	640	3130
eval_metric	r2_metric	r2_metric
early_stopping_rounds	100	200

**Table 10:** Model parameters for biological age prediction with filtered dataset.

parameter	male	female
lambda_l1	1	55
lambda_l2	56	34
learning_rate	0.0052	0.0427
max_depth	5	10
min_child_weight	0	8
min_data_in_leaf	20	30
n_estimators	4075	1550
num_leaves	2510	460
n_jobs	−1	−1

### Evaluation metrics

In the biological age prediction experiment, we use R-square, MAE, MSE, and RMSE as the metrics to evaluate our prediction results.

*R*^2^, also known as the coefficient of determination, is used to measure how well the predicted values fit the actual values. Its value ranges from 0 to 1, with values closer to 1 indicating that the model’s predictions are more accurate:


$$R^{2}=1-\frac{\sum_{i=1}^{n}(y_{i}-{\hat{y}}_{i}{)}^{2}}{\sum_{i=1}^{n}(y_{i}-\bar{y}{)}^{2}}$$

Where *y*_*i*_ is the actual value, ${\hat{y}}_{i}$ is the predicted value, and $\bar{y}$ is the mean of the actual values.

MAE (Mean Absolute Error) measures the average absolute error between the predicted and actual values. Its calculation formula is:


$$MAE=\frac{1}{n}\sum_{i=1}^{n}|y_{i}-{\hat{y}}_{i}|$$

MSE (Mean Squared Error) measures the squared error between the predicted and actual values. Its calculation formula is:


$$MSE=\frac{1}{n}\sum_{i=1}^{n}(y_{i}-{\hat{y}}_{i}{)}^{2}$$

RMSE (Root Mean Squared Error) is the square root of MSE. Its calculation formula is:


$$RMSE=\sqrt{\frac{1}{n}\sum_{i=1}^{n}(y_{i}-{\hat{y}}_{i}{)}^{2}}$$

Standard Deviation (SD) measures the dispersion of a dataset. Its calculation formula is:


$$SD=\sqrt{\frac{1}{n}\sum_{i=1}^{n}(y_{i}-\bar{y}{)}^{2}}$$

### Biological age prediction experiment

#### Experimental process.

In the biological age prediction experiment, our primary approach is to develop a machine learning model to predict residual life expectancy. This model takes users’ physical examination and questionnaire data as input and outputs their predicted remaining lifespan. We then use a conversion formula to translate this predicted lifespan into biological age. In this experiment, we first apply commonly used machine learning models to compare their performance on the biological age prediction task. Based on the results of this initial experiment, we will select a model for further testing. We will then use datasets obtained through MICE imputation and filtering to evaluate the effectiveness of these two methods of handling missing values. This comparison will help determine which method to use for missing value processing in subsequent experiments.

#### Comparison of different machine learning models experiment.

**Fig 15:**
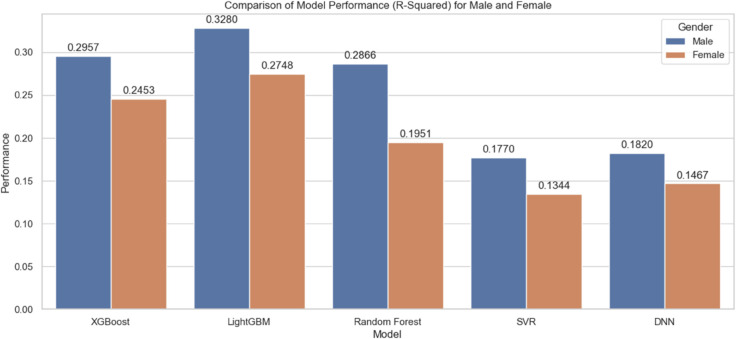
Comparison of different residual life prediction models.

We conducted experiments using currently popular machine learning models to determine the final model for biological age prediction. In this experiment, we used the data obtained through MICE imputation, and the experimental results are shown in Fig 15.

As shown in the Fig 15, the LightGBM model provides the best prediction performance. Therefore, we will use the LightGBM model for the subsequent experiments. We believe the poor performance of the DNN model is primarily due to the large number of features in our dataset and the relatively small number of records, making it challenging to effectively train the DNN model.

#### Experiment comparing different methods for handling missing values.

We employed two different methods for handling missing values to generate the final datasets for model training. Here, we designed a comparative experiment to evaluate the effectiveness of these two methods for this task. The goal is to determine the most suitable method for handling missing values in our subsequent experiments.

#### Dataset Obtained by Imputation.

In this experiment, we use the complete dataset obtained by imputation to build a machine learning model for residual life prediction, which is then converted into biological age.

**Fig 16:**
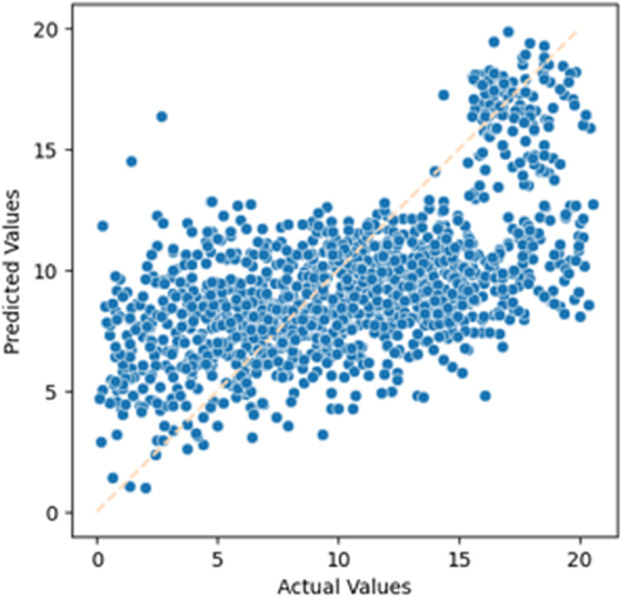
Residual life prediction using the imputed dataset (Male).

**Fig 17:**
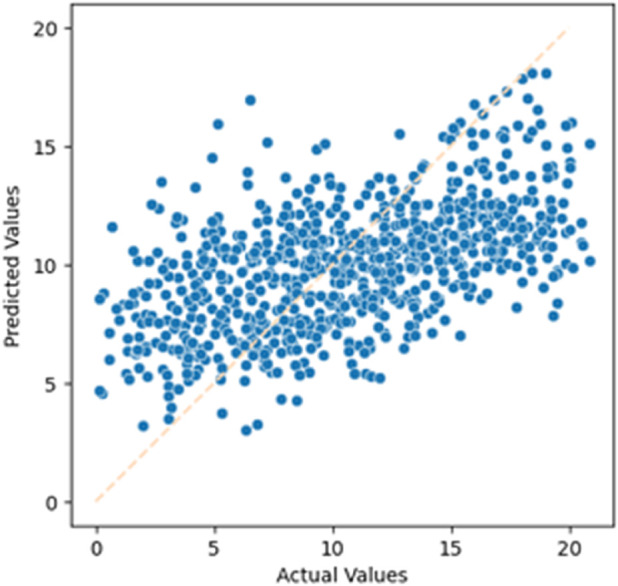
Residual life prediction using the imputed dataset (Female).

In the experimental results, we used scatter plots to represent prediction performance. The results are shown in Figs 16 and 17. These two figures illustrate the residual life prediction performance on the test set of the imputed dataset. The horizontal axis represents the actual residual life values, while the vertical axis shows the predicted residual life values generated by the LightGBM model trained on the training set. The closer the blue points are to the light orange dashed line, the better the model’s performance. The performance of this model under various metrics is summarized in Table 11, with R-squared being our primary focus.

**Table 11:** Residual life prediction metrics using the imputed dataset.

Metrics	Male	Female
R-squared	0.3280	0.2748
MAE	3.3917	3.4766
MSE	17.583	17.879
RMSE	4.1933	4.2283

#### Dataset Obtained by Filtering.

**Fig 18:**
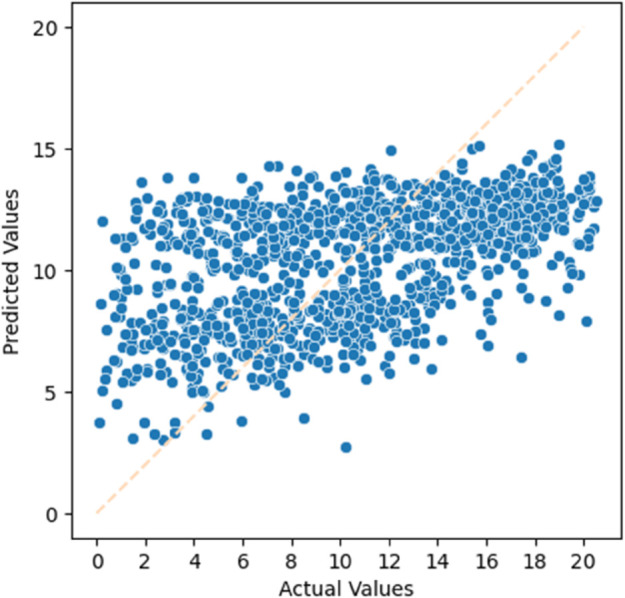
Residual life prediction using the filtered dataset (Male).

**Fig 19:**
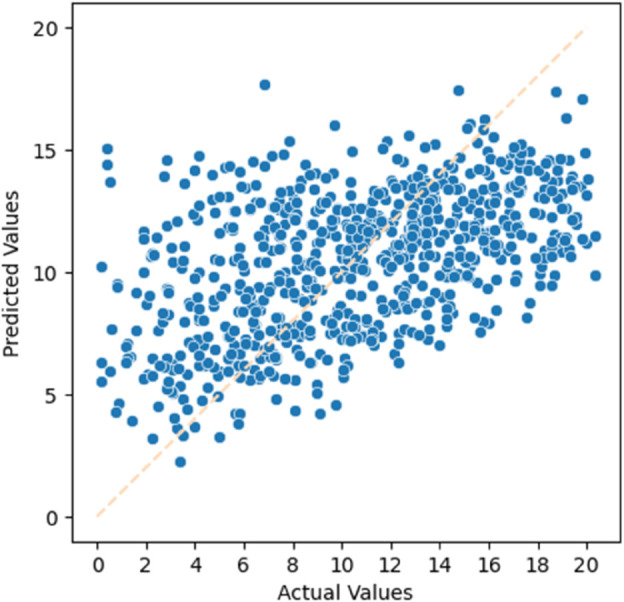
Residual life prediction using the filtered dataset (Female).

The experimental results using the filtered dataset are shown in Figs 18 and 19. These two figures represent the residual life prediction performance in the test set of the filtered dataset. The evaluation metrics for the experimental results are presented in Table 12.

**Table 12:** Residual life prediction metrics using the filtered dataset.

Metrics	Male	Female
R-squared	0.2452	0.2435
MAE	3.6915	3.4752
MSE	19.843	18.650
RMSE	4.4545	4.3186

#### Analysis and Conclusion.

By comparing the two different missing data handling methods, we can observe that the dataset obtained through MICE imputation outperforms the dataset obtained through filtering in terms of various evaluation metrics. Therefore, in the subsequent experiments, we will choose to use the dataset obtained through MICE imputation for further analysis.

#### Analysis of experimental results.

From the experimental results, we can observe that by selecting different models, the LightGBM model we chose improved the R-squared metric by 11.40% compared to the general XGBoost model. Additionally, by choosing different data imputation methods, the MICE imputation method improved the R-squared metric by 23.35% compared to the rule-based data filtering method.

In this experiment, by examining the scatter plots, we can see that our biological age prediction method has achieved certain results. A more detailed analysis of the performance will be conducted in the following experiments.

### Biological age assessment experiment

#### Experimental process.

In the biological age assessment experiment, our main approach is to use the Kaplan-Meier (K-M) estimator from survival analysis to evaluate the proposed biological age. We use the dataset obtained through the MICE imputation method and construct the K-M estimator on its test set to determine whether the biological age we propose correctly classifies different population groups.

#### Experimental results.

**Fig 20:**
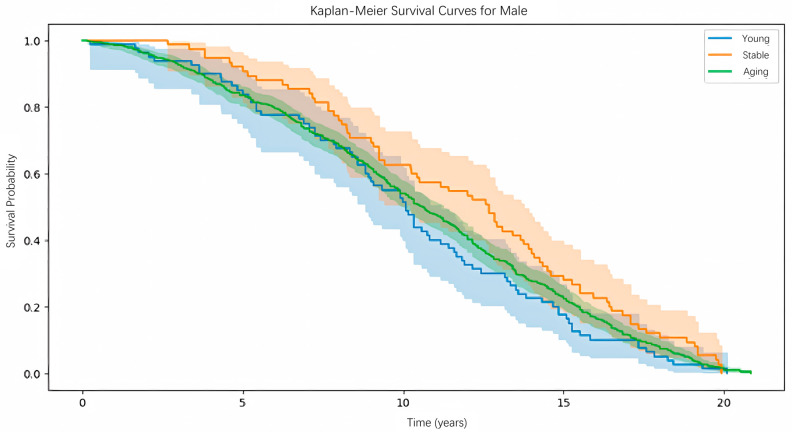
K-M curve evaluation of biological age prediction model (Male).

The experimental results for males are shown in Fig 20. In the survival analysis results for males, the three survival curves for the Young, Stable, and Aging groups are clearly distinct and arranged from top to bottom in the expected order. The difference among the three survival distributions was statistically significant (log-rank test, *p* < 0.001). Taking the blue line as an example, after 10 years following a health check-up, individuals classified in the “Young” group based on our proposed biological age have the highest survival probability, followed by those in the “Stable” and “Aging” groups. From the figure, it is clear that the three curves representing different biological ages show a high level of differentiation, indicating that biological age provides a reasonably accurate assessment of an individual’s health status.

**Fig 21:**
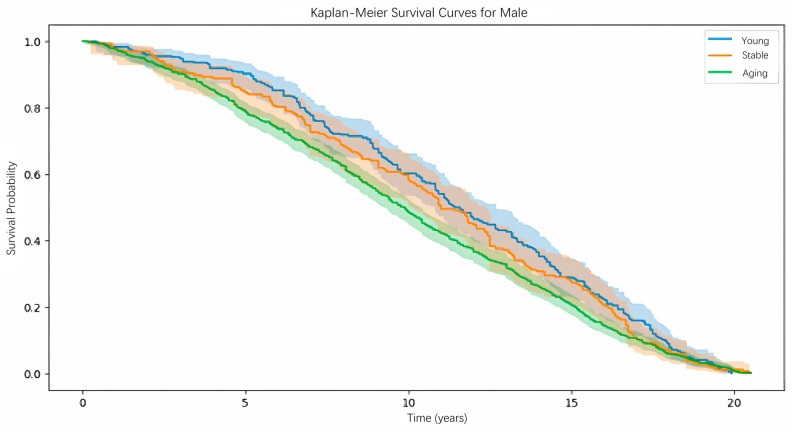
K-M curve evaluation of biological age prediction model (Female).

The experimental results for females are shown in Fig 21. As seen, the order of the three survival curves does not match our expectations, with the sequence being Stable, Aging, and Young from top to bottom. We suspect this discrepancy is due to the small number of samples in the Young group for females. After splitting by gender, training, validation, and test sets, as well as categorizing by aging status, the number of samples in the Young group for females is fewer than 80, which may lead to more fluctuating results. However, the Stable and Aging groups show clear distinctions, similar to the male group. Overall, the difference among the three groups was not statistically significant (log-rank test, *p*>0.05). To improve the prediction accuracy for young females’ biological age in the future, we should include more data from younger females in the training set.

### Exploratory experiment on features influencing biological age

#### Experimental process.

In the exploratory experiment on features influencing biological age, we aim to identify the features most relevant to biological age prediction—essentially those most indicative of aging—through feature analysis. We organize these features and compare them with findings from literature on health impact factors to determine whether the key influences our biological age prediction model identifies align with current knowledge. Finally, we apply SHAP and PCC methods to provide health check participants with actionable insights for potential life extension.

In our study, we identified aging-related biomarkers by listing the average gain of each feature within the trees of the LightGBM model. We then ranked these features to determine the top ten that most significantly impact biological age prediction.

In comparison with other studies, we referenced a previous study [22], which also compiled factors affecting health. Additionally, we gathered data on the top ten causes of death in Taiwan [23]. In the subsequent experimental results, these are listed alongside the top ten factors most impacting biological age in our research.

#### Experimental results.

The top ten factors impacting biological age identified in this study are compared with the two previously mentioned data sources, as shown in Table 13.

**Table 13:** Summary of aging-related biomarkers.

Top 10 Biomarkers	This Study		Previous Research [22]	Causes of Death [23]
	Male	Female		
Urinary Cast Deposition	•	•		•
Lactate Dehydrogenase	•	•	•	•
Albumin Level	•	•	•	•
Alkaline Phosphatase	•	•	•	•
Rheumatoid Factor	•			
Alpha-Fetoprotein	•			•
Hematocrit	•		•	
Total Iron-Binding Capacity	•			
Carcinoembryonic Antigen	•	•		•
Platelets	•	•	•	
Red Blood Cells		•	•	
History of ENT Surgery		•		
Age of Menopause		•		

*Note: A ‘• ’ indicates that the biomarker was identified as a top-ten factor in the corresponding study or list.*

From the experimental results, it can be observed that the primary factors affecting biological age in our study have a 69.23% overlap with either the findings from prior research [22] or Taiwan’s top ten causes of death [23]. This overlap indicates that our method for calculating biological age demonstrates a reasonable degree of accuracy. Additionally, factors that do not overlap may represent new findings that could influence longevity and warrant further attention in future healthcare practices.

### Experiment on exploring the impact of healthcare recommendations on individuals using SHAP and PCC

#### Experiment process.

Based on the concept outlined in Fig 14, we designed the following experimental workflow. First, we apply the SHAP method to identify the top three longevity-reducing factors in a given health check record. For each of these three factors, we calculate the three features with the highest correlation (i.e., PCC). Next, we regress these three longevity-reducing factors and their associated high-correlation features to the average level in the dataset. After updating the record with the revised factors, we use the model to predict the new residual life and compare it with the original residual life prediction to assess whether the life expectancy has been extended. Furthermore, when the absolute change in residual life is less than 0.5 years, we consider the change in residual life as insignificant.

#### Experiment results.

**Fig 22:**
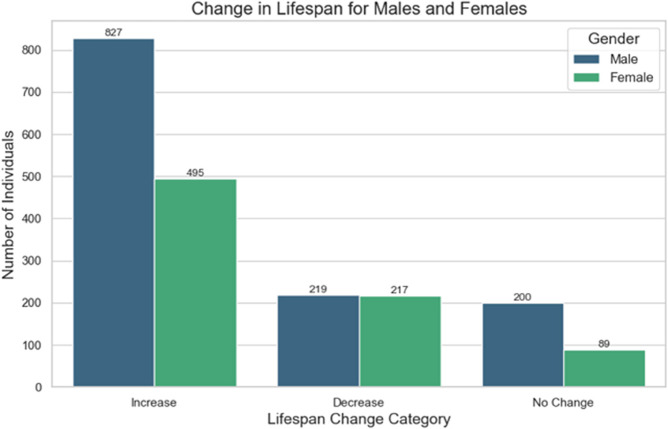
PCC combined with SHAP experiment results.

The experiment results, as shown in Fig 22, indicate that regardless of gender, after regressing the top three life-shortening factors and their highly correlated factors to the average level of the same age group, 64.58% of individuals saw an improvement in their remaining life expectancy. 14.19% of individuals’ life expectancy remained unchanged, while 21.30% experienced a reduction in their life expectancy.

We believe that for 14.19% of individuals, even though the impact of the top three life-shortening factors has been improved, the factors leading to their death might be unrelated to the top three death factors. Therefore, it is normal that their lifespan remains unaffected. However, for 21.30% of individuals, even after improving the top three life-shortening factors, a reduction in lifespan was observed. We believe this suggests that the current model still has certain limitations, and we can explore the reasons for this phenomenon in future studies.

## Conclusion and future work

### Conclusion

This study proposes a machine learning-based biological age prediction model that evaluates an individual’s physiological aging status through health examination data and residual life estimation. Compared to traditional methods that rely on a single biomarker, our model employs the LightGBM algorithm combined with the SHAP interpretability tool, enhancing both prediction accuracy and interpretability. Experimental results show that, using the MICE imputation method to address missing data, the accuracy of biological age predictions improves by 23.35% compared to using data filtering methods. The prediction results were validated through survival analysis (via the Kaplan-Meier estimator), demonstrating the effectiveness of biological age in distinguishing individuals of varying health conditions. This finding not only supports the model’s reliability but also indicates that biological age can serve as a valuable metric for assessing individual health status and aging progression.

By analyzing the biomarker factors affecting the model’s predictive performance, we identified the top ten key biomarkers related to aging for both men and women. Some of these biomarkers overlap significantly with Taiwan’s leading causes of death and findings from previous research, which further validates the model’s potential application value.

Through the integration of SHAP and PCC analyses, the model not only provides predictions but also offers clear interpretive pathways and health intervention recommendations aimed at slowing the aging process. In our experimental setting, these health recommendations demonstrated a notable effect in enhancing expected longevity for 64.58% of individuals. This interpretive mechanism not only improves the model’s transparency but also establishes a foundation for future personalized health interventions based on biological age.

### Future work

In the course of our research, several areas warrant further exploration:

- Medical health examination data often contains missing values, and how to address these missing values can significantly impact the development of biological age prediction models. Future work could explore different imputation methods, including those specifically designed for medical data.
- Currently, the proposed biological age predictions undergo validation with the K-M estimator, which requires a complete dataset. Developing a biological age prediction model without imputation and identifying a method to validate it could be an interesting direction for future research.
- In the K-M estimator validation, the results for the Young group in females only aligned with expectations when combined with the Stable group. This discrepancy could be due to insufficient sample size. Exploring ways to validate biological age predictions with limited samples is a potential area for future work.
- For interpretability, we combined PCC and SHAP to offer actionable recommendations to health check participants for lifespan extension. However, due to PCC’s inability to process categorical variables, such variables were excluded. Finding methods to determine if a categorical variable is highly related to a specific factor influencing lifespan could enhance this approach.
